# Inactivation of the Lateral Entorhinal Area Increases the Influence of Visual Cues on Hippocampal Place Cell Activity

**DOI:** 10.3389/fnsys.2017.00040

**Published:** 2017-05-29

**Authors:** Kristin M. Scaplen, Rohan N. Ramesh, Negin Nadvar, Omar J. Ahmed, Rebecca D. Burwell

**Affiliations:** ^1^Department of Neuroscience, Brown UniversityProvidence, RI, United States; ^2^Department of Biomedical Engineering, University of MichiganAnn Arbor, MI, United States; ^3^Department of Psychology, University of MichiganAnn Arbor, MI, United States; ^4^Department of Cognitive, Linguistics and Psychological Science, Brown UniversityProvidence, RI, United States

**Keywords:** hippocampus, theta, entorhinal cortex, navigation, landmarks, objects

## Abstract

The hippocampus is important for both navigation and associative learning. We previously showed that the hippocampus processes two-dimensional (2D) landmarks and objects differently. Our findings suggested that landmarks are more likely to be used for orientation and navigation, whereas objects are more likely to be used for associative learning. The process by which cues are recognized as relevant for navigation or associative learning, however, is an open question. Presumably both spatial and nonspatial information are necessary for classifying cues as landmarks or objects. The lateral entorhinal area (LEA) is a good candidate for participating in this process as it is implicated in the processing of three-dimensional (3D) objects and object location. Because the LEA is one synapse upstream of the hippocampus and processes both spatial and nonspatial information, it is reasonable to hypothesize that the LEA modulates how the hippocampus uses 2D landmarks and objects. To test this hypothesis, we temporarily inactivated the LEA ipsilateral to the dorsal hippocampal recording site using fluorophore-conjugated muscimol (FCM) 30 min prior to three foraging sessions in which either the 2D landmark or the 2D object was back-projected to the floor of an open field. Prior to the second session we rotated the 2D cue by 90°. Cues were returned to the original configuration for the third session. Compared to the Saline treatment, FCM inactivation increased the percentage of rotation responses to manipulations of the landmark cue, but had no effect on information content of place fields. In contrast, FCM inactivation increased information content of place fields in the presence of the object cue, but had no effect on rotation responses to the object cue. Thus, LEA inactivation increased the influence of visual cues on hippocampal activity, but the impact was qualitatively different for cues that are useful for navigation vs. cues that may not be useful for navigation. FCM inactivation also led to reductions in both frequency and power of hippocampal theta rhythms, indicative of the loss of functionally important LEA inputs to hippocampus. These data provide evidence that the LEA is involved in modulating how the dorsal hippocampus utilizes visual environmental cues.

## Introduction

Previous work from our lab examined the effects of manipulating two-dimensional (2D) landmarks and objects on hippocampal place fields. Landmarks were operationalized as large 2D cues on the floor of a foraging arena adjacent to the walls, and objects were operationalized as small 2D cues placed away from the walls. We demonstrated that 2D landmarks can serve as orienting cues during navigation. In addition, our findings suggested that 2D objects are processed differently by the hippocampus, perhaps as non-stationary cues not suitable for navigation but available for associative learning (Scaplen et al., 2014). A remaining question is how other regions in the hippocampal system influence processing of these cues. The entorhinal cortex provides the primary cortical input to the hippocampus, and recent data suggest the lateral subdivision is involved in processing both spatial and non-spatial aspects of an environment. Thus, it is reasonable to suggest the entorhinal cortex modulates how landmarks and objects are utilized by the hippocampus.

The entorhinal cortex is divided into medial entorhinal area (MEA) and lateral entorhinal area (LEA) based on morphological differences (Krieg, 1946a,b; Blackstad, 1956). The MEA has a well-established role in path integration and spatial processing (Moser and Moser, 1998, 2008; Fyhn et al., 2004; Parron and Save, 2004a,b; Parron et al., 2004; Hafting et al., 2005; Van Cauter et al., 2013), whereas data suggests the LEA is involved in processing both spatial and nonspatial information. Cortical and subcortical connections are consistent with this view (Burwell and Amaral, 1998; Agster and Burwell, 2009, 2013; Agster et al., 2016; Tomás Pereira et al., 2016). Damage to the LEA induces profound deficits in item recognition memory, but also mild deficits in contextual recognition memory (Hunsaker et al., 2013). In addition, interference with the signaling of the glycoprotein reelin in LEA impairs performance in place navigation tasks (Stranahan et al., 2011). Further, LEA c-fos expression and lesion studies identify a specific role in linking objects to a particular context (Wilson et al., 2013).

Neural activity in the LEA is correlated with processing of objects and spatial contexts. LEA, but not MEA, firing field positions were influenced by the presence and movement of three-dimensional (3D) objects (Deshmukh and Knierim, 2011). A separate class of LEA neurons fire at discrete locations in relation to the prior position of an object (Tsao et al., 2013). 3D objects also influence the size and number of distal CA1 place fields (Burke et al., 2011). CA1 receives direct projections from LEA. Together, these data suggest that LEA plays a role in processing objects, their locations, and the memories of previously experienced object-location associations.

Is LEA also involved in processing spatial and nonspatial information about 2D visual stimuli? The present study addressed the hypothesis that LEA modulates hippocampal processing of 2D landmarks and objects. LEA was inactivated using fluorescently conjugated muscimol (FCM), a potent GABA_A_-agonist that rapidly and reversibly suppresses neural activity allowing within subject comparisons across floor conditions and drug treatments (Allen et al., 2008; Scaplen et al., 2014). We predicted that when LEA was inactivated, the manipulation of visual cues would have less control over hippocampal processing, thus LEA inactivation would decrease the likelihood of coherent rotations in the Landmark Floor condition and decrease remapping responses in the Object Floor conditions. Unexpectedly, we found that LEA inactivation increased rotation responses to manipulations of the landmark cue and increased information content of place fields in the presence of the object cue.

## Materials and Methods

### Subjects

Six male Long Evans rats (Charles River Laboratories, Wilmington, MA, USA) were 2–4 months old (275–350 g) at the time of implantation. Rats were housed individually, before and after surgery, in large Plexiglas^®^ cages and kept on a 12 h light/12 h dark schedule. All testing occurred during the light phase. Prior to behavioral training, rats were brought to 85%–90% of *ad libitum* weight with *ad libitum* access to water. This study was carried out in accordance with NIH guidelines for the care and use of rats in research. The protocol covering these experiments was approved by the Brown University Institutional Animal Care and Use Committee approvals.

### Surgery, Electrode Preparation and Localization

All microdriver assemblies were built in-house and encased within a plastic funnel for protection. Each assembly consisted of eight independently driveable tetrodes constructed from four 12 μm nichrome wires twisted together (California Fine Wire Company, Grover Beach, CA, USA). Prior to surgery, electrode tips were cut and plated with platinum chloride (Sigma-Aldrich, St. Louis, MO, USA) to reduce impedances to 100–300 kΩ at 1 kHz.

All surgeries were performed under aseptic conditions. Thirty minutes prior to the beginning of surgery, rats were given the anticholinergic glycopyrrolate (0.5 mg/kg SC), the antiepileptic diazepam (2 mg/kg I.P.), analgesic butorphanol tartrate (0.5 mg/kg SC) and carprofen (5.0 mg/kg SC). Rats were deeply anesthetized with vaporized isoflurane (2%) and secured in a stereotaxic apparatus using blunt ear bars. Each surgery required two ipsilateral craniotomies for the implantation of a microdrive assembly above dorsal hippocampus and a guide cannula directed at LEA. The microdrive assembly was oriented at a 16° angle in the caudal plane (AP 3.126 mm, ML 2.5–3.0 mm relative to bregma and DV 2.3–2.4 mm relative to cortex) so the assembly would not interfere with the infusion cannula. Tetrodes extending from the microdrive assembly were inserted above the right hippocampus in one circular bundle (2–2.5 mm diameter). The infusion cannula was implanted at a 12° angle in the lateral plane (AP 7.6 mm, ML 5.0 mm relative to bregma and DV 6.0 mm relative to cortex). The guide cannula was implanted and secured to the skill prior to the microdrive assembly implantation. Jeweler’s screws and bone cement were used to secure the microdrive to the skull after implantation. One or two screws in the skull of the contralateral hemisphere were connected to the microdrive ground.

At the end of the experiment, rats were anesthetized, electrode tip placements were marked with a small lesion and LEA was infused with FCM to determine the spread of the GABAa agonist. Thirty minutes later, rats were euthanized with a lethal dose of Beuthanasia-D (100 mg/kg, i.p., Merck and Co. Inc., Whitehouse Station, NJ, USA) and perfused with 0.1 M Phosphate Buffer in 0.9% Saline, 0.9% Saline and 4% Paraformaldehyde to optimize fluorescence visualization. Brains were extracted and prepared for fluorescence visualization and histology.

### Two-Dimensional LEA Flat Map Construction

Contours of Nissl stained material were drawn for a 1:2 series of 60 μm sections ipsilateral to the implanted cannula and electrode implantation site at low magnification using a light microscope and attached drawing tube. Borders of LEA were outlined and individual electrode tracts and/or lesions were drawn. Layer 2 of LEA was unfolded into 240 square pixels. Images of fluorescence spread were taken at 5×, stitched together and aligned with companion Nissl sections to identify the location of FCM spread on the 2D flat map.

### Behavioral Apparatus

**Figure 1:**
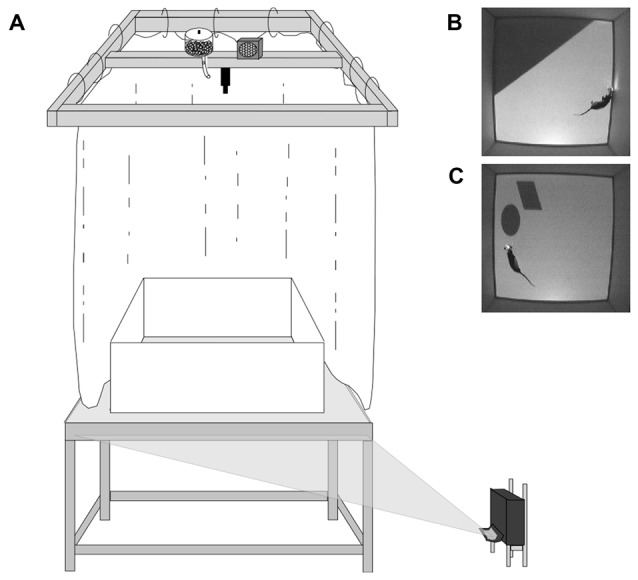
Behavioral apparatus and cue conditions. **(A)** Schematic of Floor Projection Maze. Floor cues are projected to the Plexiglas^®^ maze floor using a short throw projector. The maze was completely enclosed by a white curtain, thereby masking any extramaze visual cues. A speaker and automatic food dispenser were located directly above the maze, near the video camera; the speaker played 70 db white noise, which served to mask auditory cues. **(B)** Top down view of an animal in the Landmark Floor Condition. **(C)** Top down view of an animal in the Object Floor Condition. Figure adapted from Scaplen et al. (2014).

The behavioral apparatus consisted of a Floor Projection Maze and a 1 m square open field arena with modular white Plexiglas^®^ walls (46 cm height, 0.6 cm thick) resting on top (Figure 1A; Furtak et al., 2009; Jacobson et al., 2014). As previously described this apparatus was a custom built 112 × 147.3 × 76 cm aluminum frame tabletop (80/20, Inc., Columbia City, IN, USA) holding a 1.27 cm thick top of clear Plexiglas^®^. The Plexiglas^®^ top was covered by flexible fabric projection screen material (Dual Vision Fabric, Da-Lite Screen Company, Warsaw, IN, USA) protected by a thin protective Plexiglas^®^ floor, which allowed for rear projection of 2D images to the maze floor (ultra-short throw projector: NEC WT615, NEC Display Solutions, Ltd.).

Three computers interfaced the Floor Projection Maze: one for tracking, one for data acquisition and one for behavioral control. CinePlex Digital Video Recording and Tracking System (Plexon, Inc., Dallas, TX, USA) with a Stingray™ camera (640 × 480 resolution, 40 frames per second) interfaced with a computer running Windows XP or Windows 7 was used for tracking LEDs. The digital video camera was positioned 140 cm above the maze floor and provided a live-image aerial view of the chamber. Behavioral control, including dropping of pellets and control of screen projection to the floor of the maze, was accomplished by a custom program written in MedState Notation and running on a Windows XP or Windows 7 under the control of MED-PC IV (Med-Associates, Inc., Burlington, VT, USA).

### Behavior and Recording Procedures

Prior to implantation, rats were trained to collect randomly scattered 45 mg dustless pellets (BioServ, Frenchtown, NJ, USA) for two or three 10-min sessions in an 81 cm^2^ arena located in a separate behavior room. The arena comprised three white Plexiglas^®^ walls and either one black Plexiglas^®^ wall or no wall on the south side (46 cm tall, 0.6 cm thick) and a white Plexiglas^®^ floor. The arena was not encircled by curtains, which allowed the animal to have visual access to distal extramaze cues. By the end of the second or third session, all rats were continually exploring and foraging the entirety of the arena.

There were two cue conditions in this experiment (Figures 1B,C), the Landmark Floor condition and the Object Floor condition. The Landmark Floor condition consisted of a projected gray floor and a dark gray triangle that occupied approximately 1/3 of the maze floor (2080 cm^2^) in the northeast corner. The Object Floor condition consisted of a gray floor with a dark gray polygon (345 cm^2^) and an ellipse (355 cm^2^) located in the northeast corner of the maze. These objects were located 5 cm from each other and approximately 4 cm from the wall. In both conditions, the walls were opaque white.

Following 5–7 days of postsurgical recovery, rats received additional habituation sessions in the recording apparatus during which time no recordings were obtained. In each session, the rat collected scattered pellets that dropped at randomly selected intervals of 10, 15 or 17 s for one 10-min session per cue condition per day, until they were habituated to the recording apparatus, automated food dispenser, and white noise. Rats were transported to and from the recording room during the habitation phase and experimental phase in a black rubber container covered with a towel to minimize any exposure to extramaze cues. Subjects were disoriented by rotating the container on route to the maze before being placed into the recording chamber and after being removed to minimize the impact of vestibular cues. Habituation sessions were terminated when rats no longer exhibited thigmotaxic behavior and collected all dropped food pellets. Rats generally required three habituation foraging sessions in each cue condition.

Once habituated, rats were screened daily for evidence of single unit activity in their home cage located in an adjacent room. The microdrive assembly was connected to a 31-channel wireless headstage (Triangle BioSystems Inc., Durham, NC, USA) with a 2X gain headstage that passed the signal to a high-gain amplifier (total = 10,000X; Plexon, Inc., Dallas, TX, USA). Recordings were band-passed filtered between 0.8 Hz and 6 kHz for single units and between 3.3 Hz and 89 Hz for local field potentials (LFP). No LFP frequencies below 4 Hz were analyzed. The signal was then processed by a Multichannel Acquisition Processor (MAP; Plexon, Inc., Dallas, TX, USA), which allowed for real-time thresholding and waveform discrimination. Spike waveforms above a threshold set by the experimenter were time-stamped and digitized at 40 kHz for 1 ms. Tetrodes were lowered in steps of 26–53 μm or less at the end of each screening day if neuronal activity was not observed. Once hippocampal activity was observed, rats were infused with either saline or FCM and 30–45 min later were run in one of the cue conditions. Importantly, the wireless headstage was turned off during infusions, but the headstage was not unplugged from the rat.

After infusion of either saline or FCM, animals were give 30–45 min to recover before recording sessions began. Rats were given 3–10 min foraging sessions in the designated condition. The first session consisted of a standard cue configuration. The second session consisted of a 90° cue rotation in either the clockwise or counterclockwise direction. Finally, the third session consisted of the same standard cue configuration as the first session. After each 10-min foraging session, the rat was removed from the recording chamber and transported to their home cage, located outside the recording room. Again, the wireless headstage was turned off during these intersession intervals; however the rats were not unplugged in order to maintain unit activity across sessions. In between each recording session the arena was wiped down with a diluted bleach solution to eliminate olfactory cues. All data files recorded on the same day from the same animal were merged offline (PlexUtil, Plexon Inc. Dallas, TX, USA) to optimize offline unit isolation.

Two LEDs, approximately 2 cm apart located on top of the head stage were used for reflective light tracking with CinePlex (Plexon, Inc., Dallas, TX, USA). Timestamped *x* and *y* coordinates of animal position were provided in real time to a MAP (Plexon Inc., Dallas, TX, USA). The third computer controlled all behavioral equipment with a DIG-716P2 Smart Control Output Interface (Med Associates Inc., St. Albans, VT, USA) including food pellet delivery, auditory white noise, and the display of floor images with custom written software in Med PC. SortClient (Plexon Inc., Dallas, TX, USA) collected animal position, single unit data and timestamps in real time for later offline analysis.

**Figure 2:**
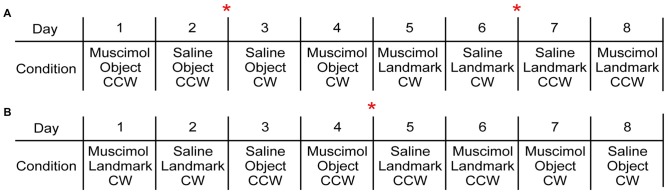
Exemplar behavioral sequences. **(A)** This behavioral sequence included 4 days of one visual cue condition in which drug condition and rotation direction were counterbalanced followed by 4 days of the second visual cue condition. Two rats were assigned to a variation of this behavioral paradigm. **(B)** This behavioral sequence decreased the number of times the electrodes were turned down as both visual cue conditions were presented within the first 4 days. Drug condition and rotation direction were counterbalanced across the following 4 days. Four rats were assigned to variations of this behavioral paradigm. The red asterisk indicates when electrodes were turned down.

With the intended experimental design, each rat experienced each floor condition four times. This allowed the order of drug treatment and rotation direction to be counterbalanced within each floor condition for each rat. The order of floor condition presentations was counterbalanced across animals (Figure 2). Electrodes were not turned down between FCM and saline treatments within the same floor condition; however no attempts were made to record from the same cell across drug treatments and therefore, no analyses were made on the same cell within or across conditions.

### Analysis

Single unit isolations were performed offline by manually sorting clusters of waveforms using Offline Sorter (Plexon Inc., Dallas, TX, USA). Sorting was performed primarily by relative peak to valley distances across each wire. The sorted clusters were subsequently screened for inter-spike intervals, and clusters showing refractory period violations were discarded from analysis. Finally, place field locations were screened and compared across cells recorded on the same tetrode to ensure individual units were not erroneously split into two separate units. Final sorted files were segmented into individual session files based on event start and stop timestamps (OfflineSorter or PlexUtil, Plexon Inc., Dallas, TX, USA) and converted to NEX files, which contained position and unit timestamps (Nex Technologies, Littleton, MA, USA). Custom written Matlab programs (Mathworks Inc., Natick, MA, USA) were used to read NEX files (readNexFile.m; author Benjamin Kraus), fix bad position coordinates, and filter data for speed (>3 cm/s; adapted from FixMyPOS.m. author R. Jonathan Robitsek). Position coordinates were obtained by averaging the coordinates of the two LEDs to obtain one position coordinate for each timestamp.

Custom Matlab programs were used to calculate spatial information content, construct speed filtered spike position, firing rate, and threshold firing rate maps as well as Gaussian smoothed (3 × 3 pixels) firing rate maps. Correlation coefficients were also calculated for spatial analysis. Place fields were identified using threshold firing rate maps that filtered the firing rate maps for pixels in which the firing rate of the cell was 3× greater than the grand average firing rate of that cell. At least five adjacent and contiguous pixels were required for the identification of a place field (Burwell and Hafeman, 2003; Scaplen et al., 2014). Spatial information content was calculated for all hippocampal cells in all three sessions per condition using smoothed data (Information Content = *P*_i_ (*R*_i_/*R*)log_2_(*R*_i_/*R*)). Where *i* is the bin number, *P*_i_ is the probability for occupancy of bin *i*, *R*_i_ is the mean firing rate for bin *i* and *R* is the mean firing rate (Skaggs et al., 1993; Markus et al., 1994). Cells were included in the analysis if spikes counts were greater than 50 and spatial information content scores were 0.25 or greater in the rotated 10-min session and either the first or second standard 10-min session. Selection criteria were similar to previous work from our lab (Scaplen et al., 2014), however, increasing the criterion for spatial information content scores to 0.50 similar to other studies (e.g., Lee and Knierim, 2007) did not change results.

In order to compare spatial firing patterns across sessions in each condition, firing rate maps for the rotated and second standard session were rotated clockwise in 90° increments and correlation coefficients were calculated in comparison to the first standard session. Place field responses within a condition were classified as rotation, remap or no change as previously described (Scaplen et al., 2014). Briefly, place fields were classified as having predictably rotated if the field rotated at least once in concert with the rotation of the relevant cue. Place fields were classified as having remapped if the field rotated or shifted to an unpredictable location with the rotation of the relevant cue. Finally, place fields were classified as having not changed if the place field remained in the same location following the rotation of the relevant cue.

LFP was also analyzed using custom Matlab routines, similar to previously described methods (Furtak et al., 2012). Here, theta oscillations on the same electrode were compared in a pairwise manner between the first session of a saline infusion experiment and the first session of an FCM infusion experiment. A total of 46 electrode pairs across five rats were analyzed. The Chronux toolbox for Matlab was used for spectral analysis of each LFP. Theta power was calculated as the mean power in the 6–10 Hz band, whereas theta frequency was calculated as the center of mass of power within the same 6–10 Hz band. For each pair, theta power and frequency were normalized to the power and frequency in the Saline treatment, and the population data was tested for statistical significance using the non-parametric Mann-Whitney-Wilcoxon (MWW) test. To control for any potential impact of speed on theta, each session was divided into 1.5-s windows, and the LFP spectrum and mean speed were computed in each window. Finally, mean theta power and frequency were then calculated only in windows where the speed was between 20–40 cm/s, and the same statistical analyses were repeated.

### Drug Preparation and Infusion Protocol

Muscimol is a potent GABA_A_-agonist that rapidly and reversibly suppresses neurophysiological activity in the targeted region. FCM (Molecular Probes, Eugene, OR, USA) is a conjugate of muscimol and the Bodipy TMR-X fluorophore that is both stable, highly lipophilic and, importantly, is shown to induce similar impairments in fear conditioning and delayed response tasks (Allen et al., 2008). FCM infusion preparation consisted of dissolving 1 mg of FCM into 1 ml of 0.01 M phosphate-buffered 0.9% saline. Dilution aliquots (1.6 mM concentration) were made and stored in a −20°C freezer until needed. The same 0.1 M phosphate buffered 0.9% saline was used for control infusions. Injections were made using a syringe infusion pump (Harvard Apparatus, Holliston, MA, USA) and a 10 μl Hamilton syringe (Hamilton Co, Reno NV, USA). The 33 g injector cannula extended 1 mm past the tip of the guide cannula when in place and was connected to the Hamilton syringe using polyethylene tubing. Animals were anesthetized using vaporized isoflurane (2%) and infused with either 0.75 μl of FCM or saline at a rate of 0.25 μl/min. Following LEA infusions, the injector cannula was left in place for 2–3 min and animals were allowed to recover for 30–45 min before beginning the task. Previous work suggests that muscimol effects are immediate and last several hours (Hikosaka and Wurtz, 1985; Krupa et al., 1999; Allen et al., 2008).

## Results

**Figure 3:**
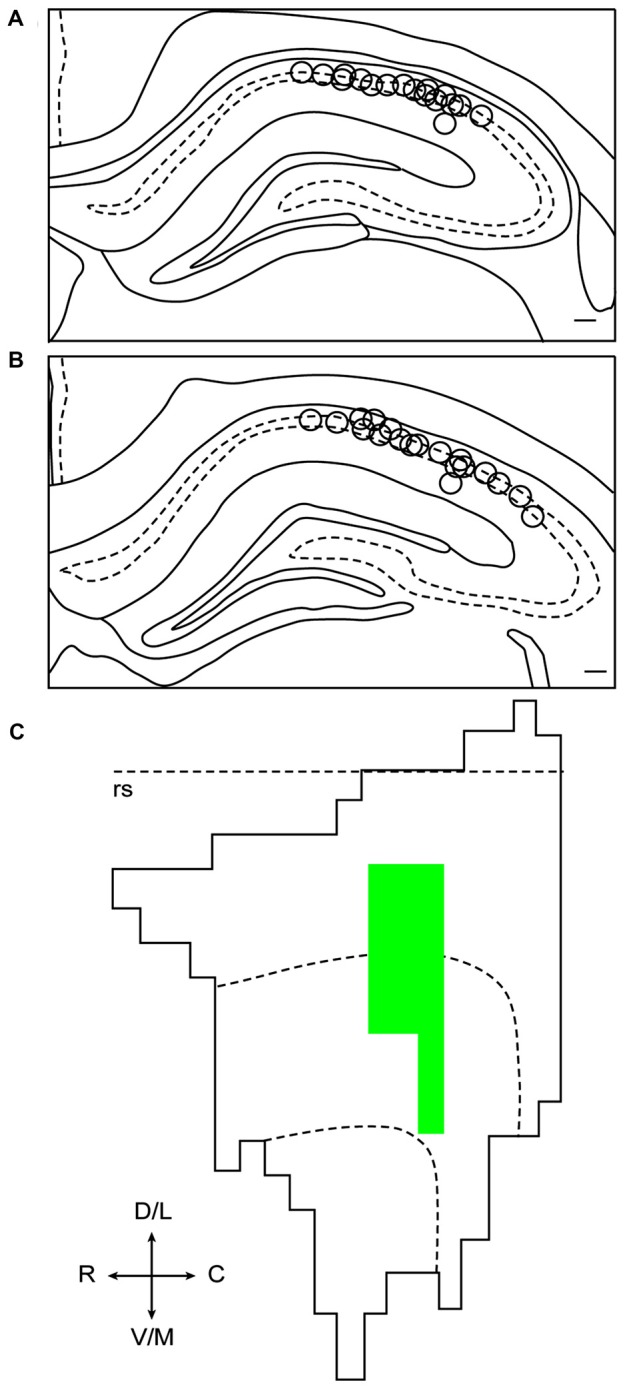
Reconstruction of electrode placements and fluorophore-conjugated muscimol (FCM) spread. **(A)** Composite oftetrode tip locations in coronal sections of dHIP between −3.20 mm and −3.36 mm relative to bregma. **(B)** Composite of tetrode tip locations incoronal sections of dHIP at between −3.60 mm and −3.80 mm relative to bregma. **(C)** Representative unfolded flat map of lateral entorhinal area (LEA). The rhinal sulcus is indicated by the dotted line. FCM spread includes portions of the lateral and intermediate bands of LEA.

A total of 260 hippocampal CA1 cells were recorded from six animals. In an effort to reduce duplicate recordings of the same cell in the same floor condition and drug treatment, electrodes were advanced in increments of at least 27 μm until new cells were evident before the presentation of a previously experienced floor condition and treatment combination. Electrodes were not advanced between recording days in different drug treatments of the same condition. A total of 67 cells were recorded in the Landmark Floor Saline treatment group; 89 cells were recorded in the Landmark Floor Muscimol treatment group; 49 cells were recorded in the Object Floor Saline treatment group and 55 cells were recorded in the Object Floor Muscimol treatment group. Of these, 24, 35, 15 and 22 cells met analysis criterion and exhibited place fields in the Landmark Floor Saline, Landmark Floor Muscimol, Object Floor Saline and Object Floor Muscimol group, respectively. Figure 3 illustrates the placements of electrodes within the CA1 subfield from which the cells included in the analysis were recorded. Table 1 shows the number of cells recorded per animal and the proportion of cells that met criterion in each condition.

**Table 1:** Percentages of cells across subjects and conditions.

	11061 (*n* = 55)	11063 (*n* = 7)	12036 (*n* = 8)	13005 (*n* = 7)	13006 (*n* = 2)	13042 (*n* = 17)
LMF-SAL	35%	0%	38%	0%	0%	12%
LMF-FCM	44%	43%	38%	0%	50%	24%
OBF-SAL	7%	43%	13%	43%	50%	18%
OBF-FCM	15%	14%	13%	57%	0%	47%

*Abbreviations: FCM, fluorophore-conjugated muscimol; LMF, landmark floor; OBF, object floor; SAL, saline*.

### LEA Inactivation Increases the Percentage of Rotations in the Landmark Floor Condition but Not in the Object Floor Condition

**Figure 4:**
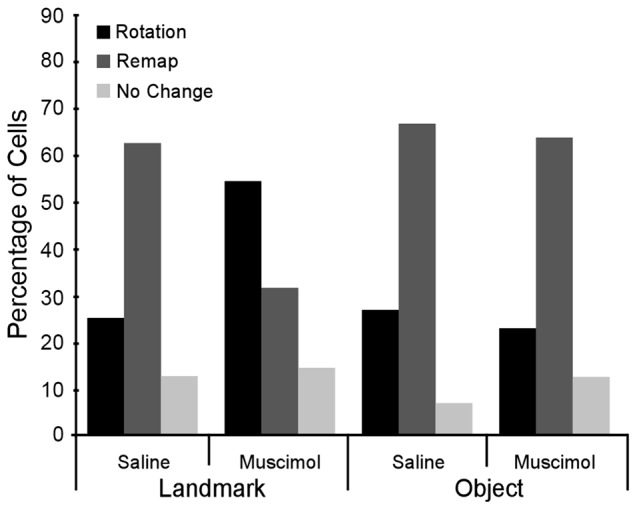
Distribution of place field responses in cue condition and drug treatment groups. Overall, cells in the Landmark Floor conditions tended to increase the percentage of rotations when LEA was inactivated. However, cells in the Object Floor condition did not change the distribution of responses when LEA was inactivated.

In the Landmark Floor Saline treatment group, the predominant response to 90° cue rotations was a remap of the CA1 place fields (Figure 4). Of the 24 cells recorded in this condition, 62.5% were classified as having remap responses, 25.0% were classified as rotation responses and 12.5% were classified as no change responses. In the Landmark Floor Muscimol treatment group, however, the predominant response was a place field rotation in concordance with the visual cue (Figure 4). Of the 35 cells recorded in this condition, 54.3% were classified as having rotation responses, 31.4% were classified as having remap responses, and 14.3% where classified as no change responses. Chi Square analysis revealed that the distribution of responses in the Landmark Floor Saline treatment group as compared to the Landmark Floor Muscimol treatment group was significantly different ($X_{\text{(2,59)}}^{\text{2}}$ = 6.034, *p* = 0.048).

In contrast, in the Object Saline and Muscimol treatment groups, the predominant response to 90° cue rotations was a remap of the CA1 place fields and a Chi Square Analysis revealed that distribution of response in these two conditions were not significantly different ($X_{\text{(2,36)}}^{\text{2}}$ = 0.517, *p* = 0.77). Of the 49 and 55 cells recorded in the Saline and Muscimol treatment groups, 66.67% and 60%, respectively, were classified as remap responses.

**Figure 5:**
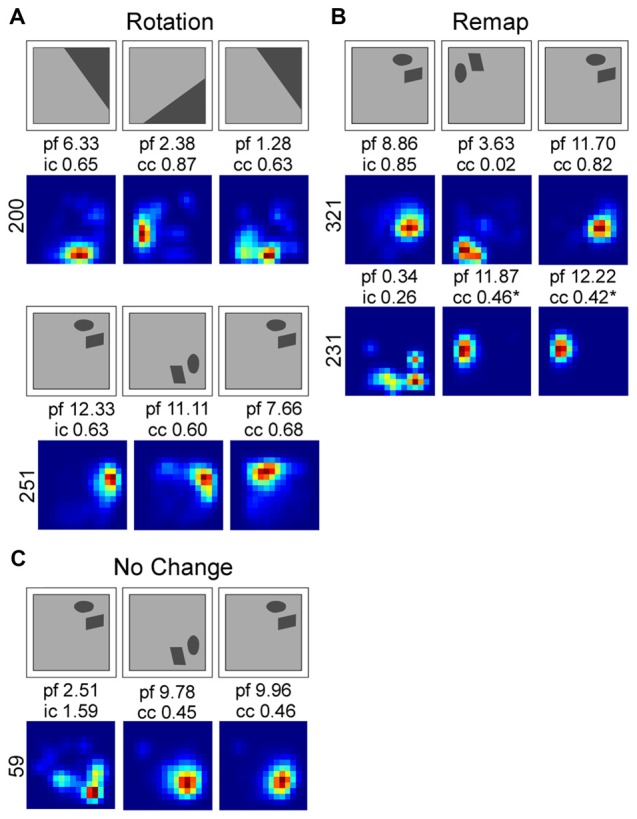
Smoothed rate maps showing exemplar place field responses. Place field responses were classified into three different categories: rotation **(A)**, remap **(B)** and no change **(C)**. Above each rate map series is a schematic showing the direction of cue rotation. Firing rate maps were smoothed for illustrative purposes. Abbreviations: peak firing field rate (p), information content score for the first standard session (i), the correlation coefficient (cc) for the rotation and second standard sessions.

Interestingly, in both Muscimol treatment groups, Landmark and Object, cells that rotated were more likely to return to their original place field location (40% and 80% respectively). In contrast only 16% of cells returned to their original place field in the Landmark Saline group and no cells rotated back to their original location in the Object Saline group. See Figure 5 for exemplar place cell responses.

### LEA Inactivations Increased the Spatial Information Content in the Object Floor Condition

**Figure 6:**
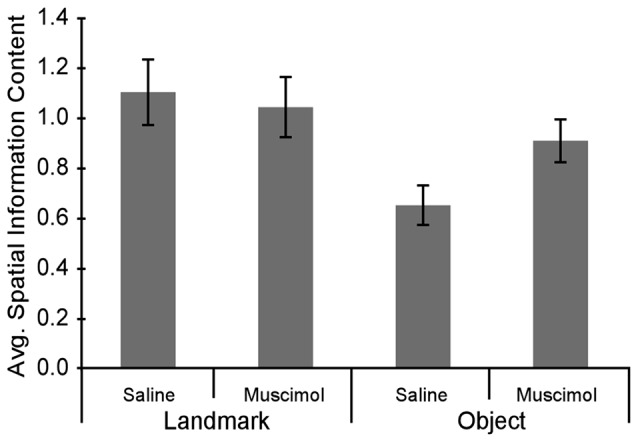
Average spatial information content in cue condition and drug treatment groups. Overall spatial information content increased in the Object Floor condition when LEA was inactivated whereas the spatial information content did not changed in the Landmark Floor condition.

**Figure 7:**
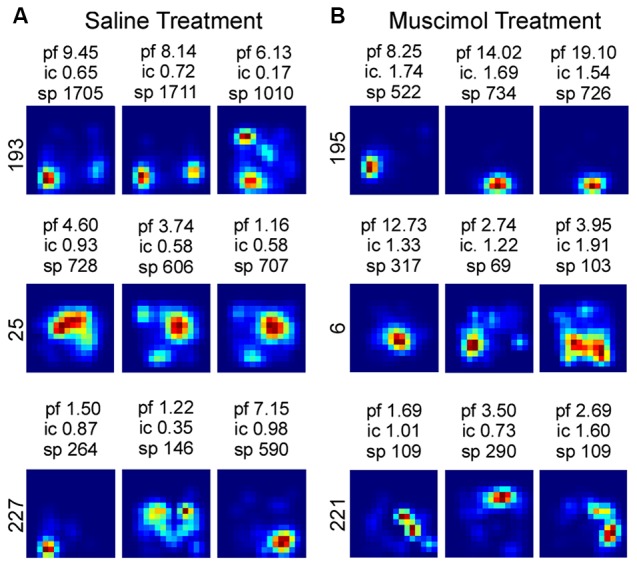
Exemplar firing rate maps of cells recorded in Object Floor condition and the associated spatial information content. The peak firing rate (pf), spatial information content (ic) and total number of spikes attributed to each cell (sp) is reported above each firing rate map. The associated cell number is listed to the left of each group of maps. Exemplar firing rate maps indicating the spatial quality of place fields recorded in the Saline **(A)** and Muscimol **(B)** treatment groups.

Although LEA inactivation did not increase the percentage of rotations in response to manipulation of the Object Floor visual cues, it did still influence neural activity; LEA inactivation increased the amount of spatial information contained in each spike (Figure 6). Spatial information content was calculated for each individual session and the three sessions in each recorded condition were averaged together. A two factor analysis of variance (ANOVA) with planned comparisons of effect of treatment in each condition (Object Floor and Landmark Floor), revealed that LEA inactivation significantly increased the spatial information content of place cells recorded in the Object Floor condition (*F*_(1,35)_ = 4.51, *P* = 0.041), but not the Landmark Floor condition (*F*_(1,58)_ = 0.11, *P* = 0.743; Figure 6). The interaction of condition and treatment was not significant (*F*_(1,94)_ = 1.60, *P* = 0.433). See Figure 7 for exemplar place cell field recorded in Saline and Muscimol treatments.

### LEA Inactivation Decreases Hippocampal Theta Frequency and Power

**Figure 8:**
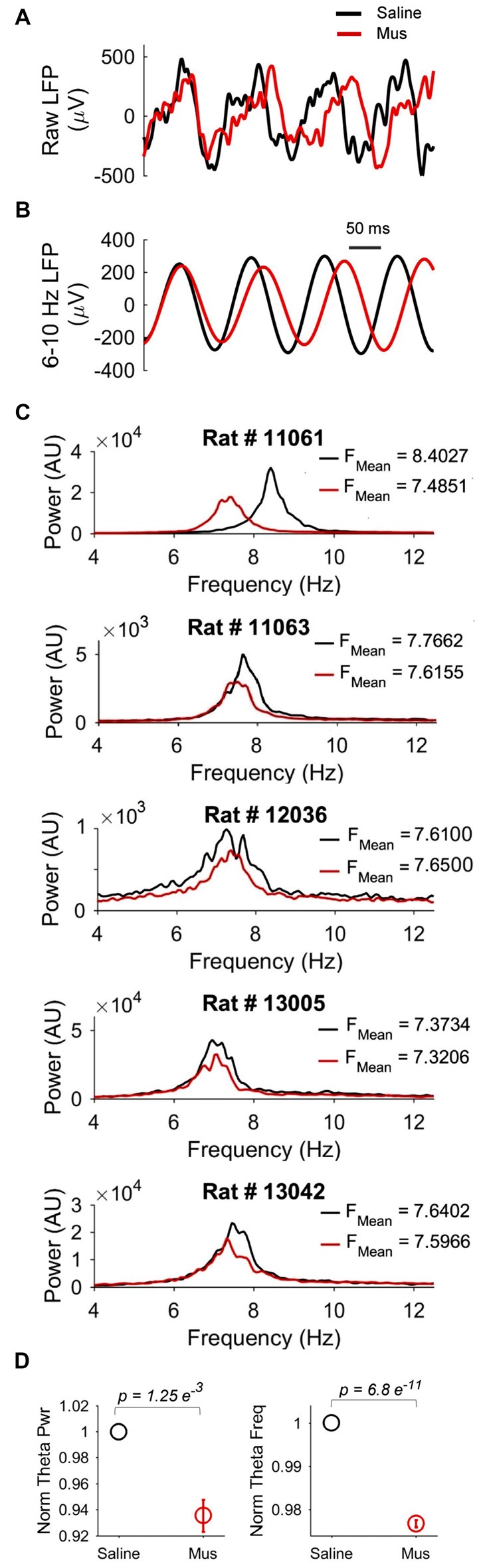
The impact of LEA inactivation on hippocampal theta oscillations. **(A)** Examples of raw local field potential (LFP) recorded from the same hippocampal electrode after saline (black) vs. Muscimol (red) infusion in LEA. Note the increased duration (and hence decreased frequency) of hippocampal theta rhythms after LEA inactivation with Muscimol. Note also the slightly decreased amplitude. **(B)** The same LFPs shown in **(A)**, but now filtered in the theta (6–10 Hz) band to highlight changes in theta frequency and power. **(C)** Examples of hippocampal LFP power spectra comparing the saline vs. Muscimol conditions in five separate rats. Note that theta-band power consistently decreased and, in four of the five rats, showed subtle shifts to the left in the Muscimol condition, indicative of lower hippocampal theta frequency with LEA inactivation. **(D)** Population averages of normalized theta power (left) and theta frequency (right) across the entire standard condition session. *p* values represent the result of the non-parametric Mann-Whitney-Wilcoxon (MWW) test, and show that both hippocampal theta frequency and power were significantly decreased with LEA inactivation.

**Figure 9:**
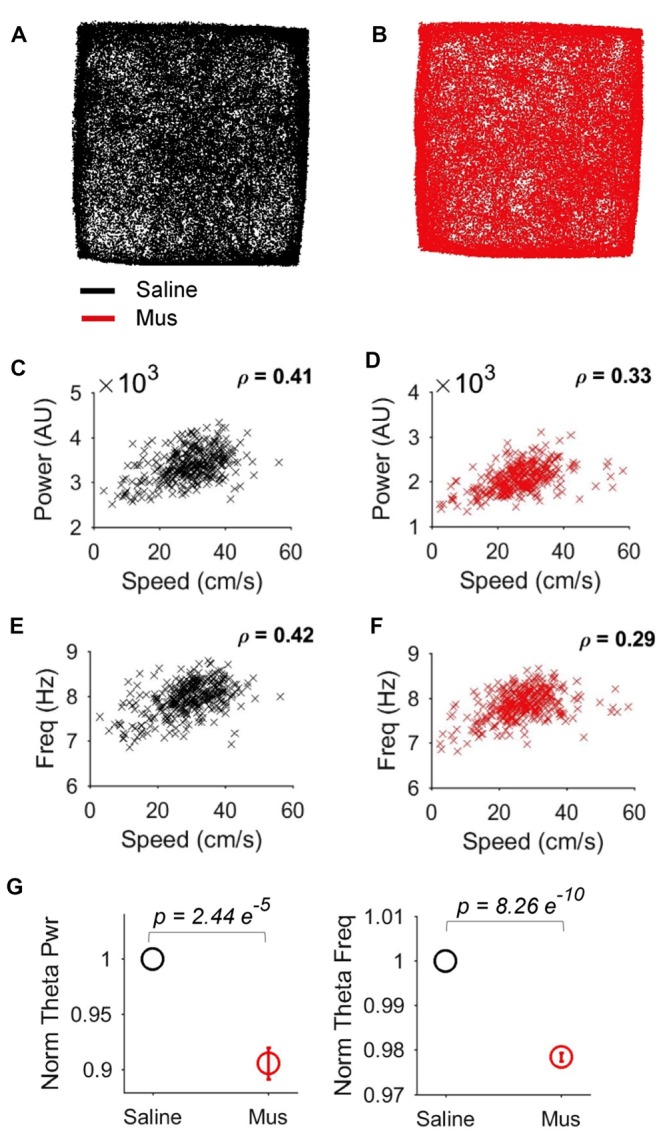
Speed-delimited decrease in hippocampal theta power and frequency. **(A,B)** Example showing similar open-field occupancy maps in the Saline **(A)** and Muscimol **(B)** conditions. **(C,D)** Relationship between running speed and hippocampal theta power in the Saline **(C)** and Muscimol **(D)** conditions. **(E,F)** Relationship between running speed and hippocampal theta frequency in the Saline **(E)** and Muscimol **(F)** conditions. **(G)** Population averages of normalized theta power (left) and theta frequency (right) in the standard condition session, but only for epochs where the rats ran between 20–40 cm/s. This speed-delimited calculation controls for the impact of speed on theta oscillations and shows that even when controlling for speed, there were significant decreases in hippocampal theta power and frequency with LEA inactivation. *p* values represent the result of the non-parametric MWW test.

We reasoned that the impact of LEA inputs on hippocampal network activity should also be reflected in the LFP. We thus compared hippocampal theta oscillations in the saline vs. Muscimol treatments. LEA inactivation with muscimol appeared to extend the duration of hippocampal theta cycles (reflecting a decrease in theta frequency), with this slowdown being visible in both the raw (Figure 8A) and theta-filtered LFP (Figure 8B). This decrease in theta frequency was also visible in the power spectra of individual LFP pairs (Figure 8C), with four out of five rats showing decreases in theta frequency, and 41/46 LFP pairs showing a decrease in theta frequency with LEA inactivation. Population data confirmed these observations, with small but significant decreases in both hippocampal theta power (*p* = 0.00125, MWW test) and hippocampal theta frequency (*p* = 6.8e-11, MWW test) after LEA inactivation (Figure 8D). Running speed is known to lead to increases in both hippocampal theta power and frequency, an observation we confirmed in our data (Figures 9A–F). To confirm that these changes in theta power and frequency were not due to changes in speed of the animals across the two treatments, we ran a further speed-controlled analysis. Only time windows with speeds between 20–40 cm/s were used to calculate mean theta power and frequency. Even when controlling for speed in this way, hippocampal theta power (*p* = 2.44e-5, MWW test) and frequency (*p* = 8.26e-10, MWW test) both decreased with LEA inactivation (Figure 9G).

## Discussion

The hippocampus processes both landmarks and objects, and the LEA exhibits object and object-location neural correlates (Deshmukh and Knierim, 2011; Deshmukh et al., 2012; Tsao et al., 2013; Scaplen et al., 2014). The present study addressed the hypothesis that the LEA modulates hippocampal processing of landmarks and objects. We predicted that when LEA was inactivated, the manipulated visual cues would have less control over the location of hippocampal place fields. Instead, we found LEA inactivation increased the influence of 2D visual cues on dorsal hippocampal place cell activity. Interestingly, LEA inactivation had different effects on rotations of landmarks vs. objects. When LEA was inactivated in the Landmark Floor condition, rotations elicited a greater number of coherent place field rotations but had no effect on information content. In contrast, when LEA was inactivated in the Object Floor condition, there was no impact on place field rotation responses, but there was an effect on the spatial information content contained in hippocampal place fields. To our knowledge, this is the first demonstration in behaving animals that the LEA modulates hippocampal activity in the presence of 2D visual cues and that LEA modulation is different for cues suitable for orientation and navigation as opposed to cues that are less useful for navigation.

Our findings are consistent with the theory that the LEA is involved in processing external (visual and nonvisual) sensory input from the environment, whereas the MEA is involved in processing internally-based, path integration computations (Deshmukh and Knierim, 2011). One interpretation of the present study is that manipulation of visual cues produced a conflict between visual cues and stationary auditory cues in the environment. Although attempts were made to mask any stationary auditory cues, we may not have been successful. Because sound can be spatially localized, manipulated visual cues would be discordant with stationary auditory cues. In cue conflict situations, one might predict place field remapping. Indeed, a large proportion of place fields remapped in the Saline treatment when the Landmark Floor was rotated. It may be that LEA is necessary for identifying cue conflict, especially when spatial cues and cues from different modalities are in conflict. Thus, when LEA was inactivated, it is possible that visual cues were more salient than the partially masked auditory cues and exerted greater stimulus control over neural activity in the dorsal hippocampus. Why would the influence of visual information be enhanced in both Landmark and Object Floor conditions when LEA is inactivated? The MEA has an established role in processing spatial context and distal landmarks (Fyhn et al., 2004; Hargreaves et al., 2005; Deshmukh and Knierim, 2011; Van Cauter et al., 2013) and its inputs are dominated by sensory input that is visual in nature, whereas the LEA is suggested to process multimodal sensory information (Kerr et al., 2007). With the diminished cue conflict in LEA, it is possible that landmarks are more likely to be classified as salient and navigationally relevant by the MEA and subsequently in the hippocampus. In other words, if the LEA is offline, the visual input to MEA from occipital regions may have relatively more influence over spatial correlates.

It is also possible that the CA1 subfield, and not the LEA, is responsible for identifying cues that are in conflict. Recent hippocampal neural data suggest that the LEA provides a local-cue derived spatial framework and the MEA provides global-cue derived spatial framework to the CA1 subfield (Neunuebel et al., 2013). Given that in previous studies, rotations of landmark floor cues predominately elicited rotation responses, these cues are likely being classified as global cues and involved in orienting the grid-cell map in MEA, whereas the remaining available cues are classified as local cues (Scaplen et al., 2014). When the Landmark Floor cues are rotated, and in conflict with stationary local cues, CA1 place cells may detect this mismatch and remap instead of rotating coherently. When the LEA is inactivated, however, the conflict between local and global spatial frameworks is diminished. Resultantly, CA1 input is dominated by landmark controlled MEA input and place cells increase rotation responses in the Landmark Floor condition.

Anatomical work indicates bilateral input from the entorhinal cortex to the hippocampus, particularly the CA1 subfield (Steward and Scoville, 1976; Wyss, 1981; van Groen et al., 2003). Although previous work reported behavior impairment as a result of unilateral LEA inactivation (Tanninen et al., 2013), we cannot discount the influence of the contralateral LEA. If LEA inactivation impairs the capability to resolve cue conflict, it may be that bilateral LEA inactivation would have resulted in even more rotations than observed with unilateral inactivation. It is also possible that the CA1 subfield is responsible for identifying a mismatch between unilateral and contralateral streams of information. In this case, when LEA is unilaterally inactivated, the conflict between local spatial frameworks might increase and bias representations toward the non-conflicting global spatial frameworks. In either case, we would predict an increase in rotation responses in the Landmark Floor condition had we bilaterally inactivated the LEA. It would be interesting, however, to directly compare the effects of unilateral with bilateral LEA inactivation on spatial representations in CA1.

Although inactivation of LEA did not elicit more rotation response in the Object Floor condition, it did influence neural activity in the hippocampus by increasing the spatial information content in hippocampal place fields. The same external sensory vs. internal processing theory described above could also explain the observed increase in spatial information content. An increase in spatial information content has been described in other regions when the spatial information available to the rat increases. For instance, recordings in LEA with little external sensory information available to the rat show diffuse firing fields and low spatial information scores (Hargreaves et al., 2005). However, when 3D objects are added to a foraging arena, LEA firing fields become more punctate and spatial information scores increase significantly (Deshmukh and Knierim, 2011). It is possible when rats are faced with conflicting cue information about an environment, the amount of spatial information rats are able to extract is diminished and therefore place cells’ spatial information content is lower. When LEA is inactivated, however, external sensory information is diminished. As a consequence, the potential of cue conflict is also diminished thereby increasing the influence of visual information on hippocampal processing, and increasing the spatial information scores of hippocampal place fields.

By contrast, LEA inactivation did not affect spatial information content in the Landmark Floor condition. This may be a ceiling effect. Alternatively, it is possible landmarks are used for spatial orientation and navigation, but not the modulation of place cell firing. Consistent with this idea, several lines of research has shown that other non-spatial qualities of an environment influence place cell firing rate, but not the location of the place field, including task demands and aversive experiences (Markus et al., 1995; Wood et al., 2000; Leutgeb J. K. et al., 2005; Leutgeb S. et al., 2005). Of course, rate remapping was seen in both conditions across sessions in this experiment, but perhaps the spatial information content is particularly influenced by the increase in sensory information the animal can extract and incorporate in a cognitive map for spatial memory. In this case, spatial information is most influenced by items or objects less likely to be used for spatial navigation or orientation, but more likely to increase the richness of a spatial memory.

One difference between previously reported data and that presented in this experiment is the distribution of place cell responses. In the Landmark Saline treatment group, the percentage of rotations was substantially different from what would be expected. One possibility is that in the current study, rats were anesthetized for infusions. This was necessary because the angle and close proximity of the cannula to the microdrive implant made it difficult to complete the infusions in awake rats. Isoflurane was selected as an anesthetic because it allows for rapid induction and recovery. It is noted that isoflurane is a potent NMDA receptor antagonist, but agreement about the nature and duration of effects is lacking (Lin and Zuo, 2011; Fidalgo et al., 2012; Cho et al., 2013). Previous work also suggests that as animals gain increased experience of cue rotations, place fields tend to decrease rotation responses (Scaplen et al., 2014). We showed that cells recorded in the first 8 days of the study showed more rotations than cells recorded in the last 8 days. In the present study, all cells were recorded on days 1–8. One possibility could be that most of the Landmark Saline cells were recorded towards the end of the experiment and thus decreased rotation responses as a result of experience. Interestingly, this is not the case. In Table 2 we show the timing of the recording of cells broken down by condition. In the Landmark Floor condition more Saline and more Muscimol cells were recorded during the last 4 days, and there were more rotations in the Muscimol condition at both time points. In any case, we argue that it is appropriate to compare drug groups within this experiment, but not across the two studies. Another possibility is that LEA inactivation elicited more rotations in the Landmark Floor condition because most of the saline data were from one or more animals that did not show rotations. This was not the case. All but one rat showed rotations in some condition. One subject (11061) had more place cells than the others, but even for this rat, there was not a disproportionate number of cells recorded in the saline condition (Table 1). Moreover, the three subjects with the largest number of cells individually showed similar responses across Landmark Floor conditions. Thus, our results cannot be accounted for by timing of recordings or overselection of one condition in a particular subject.

**Table 2:** Effects of experience on rotations.

	Landmark		Objects	
	SAL	FCM	SAL	FCM
Days 1–4				
# Cells	5	10	12	20
% ROT	0%	60%	33%	25%
Days 5–8				
# Cells	19	25	3	2
% ROT	32%	52%	0%	0%
All Days				
# Cells	24	35	15	22
% ROT	25%	54%	27%	23%

*Number of cells in each condition for first and last 4 days of recordings. Abbreviations: FCM, fluorophore-conjugated muscimol; ROT, rotation cells; SAL, saline*.

Following the discovery of grid cells in 2005, MEA became a heavily researched area of the brain. More recently, attention has been directed to the LEA, but there are open questions about the information processed by LEA and the computations performed. Our data are consistent with a proposal that MEA processes internal ideothetic information and LEA processes external sensory information (Deshmukh and Knierim, 2011). In addition, the present study provides evidence that LEA modulates hippocampal processing of landmarks and objects by integrating information from multiple modalities to guide use of sensory information for orientation and navigation. Our data also suggest that, in the absence of LEA input, the hippocampus is less affected by conflicting sensory cues. Inactivation of the LEA appeared to increase the saliency of the object cues, but did not appear to change how the objects were used with regard to orientation. Thus, LEA seems to be necessary for integrating complex multimodal sensory information whether such information is used for navigation or other purposes. When input from LEA is reduced, the most salient stimuli exert more control over hippocampal responses.

Hippocampal theta oscillations reflect integrated inputs from multiple brain regions and act as an important readout of hippocampal computations (Buzsáki, 2002). When we inactivated LEA, we observed small, but consistent decreases in hippocampal theta frequency and power. These decreases were independent of running speed. The observation that both theta power and frequency were decreased suggests that LEA inputs play a role in controlling both the power and frequency of theta oscillations, likely via the same mechanism. Under standard conditions it has been shown that both hippocampal theta power and hippocampal theta frequency increase with faster running speeds (Ahmed and Mehta, 2012), highlighting the tight relationship between theta power and speed. Thus, the changes observed here likely reflect a net decrease in synaptic inputs to the CA1 region when the LEA inputs are removed. This net decrease in excitation is likely to lead to both reduced amplitude and frequency of theta oscillations. LEA and MEA have important anatomical differences in their projections to CA1: MEA projects predominantly to cells in proximal CA1 (closer to CA3) whereas LEA projections preferentially target cells in distal CA1. Thus future studies that simultaneously record from multiple parts of the proximodistal CA1 axis will help to test the hypothesis that the changes we see in theta oscillations and information encoding will be most pronounced in distal CA1 after LEA inactivation (Knierim et al., 2006; Ahmed and Mehta, 2009; Henriksen et al., 2010). These studies would also be important in understanding the differential effect of inactivating LEA on proximal vs. distal CA1. It is possible that place cell responses to manipulations in the absence of LEA input would be more prominent in distal CA1. The bundling of electrodes in this study precluded us from identifying proximal vs. distal CA1 electrodes. Such a comparison would, however, be especially informative.

A related question is what happens to place field representations following transient MEA inactivation. Preliminary data from our lab suggests that MEA inactivation has minimal effects on place cell responses to landmark and object manipulations (data not shown). Interestingly, a recent report from Rueckemann et al. (2016) also suggests that MEA is less involved in the spatially selective responses of CA1 neurons. Rueckemann et al. (2016) reported that transient optogenetic inactivation of MEA resulted in remapping in some (approximately 30%), but not all, hippocampal place fields. The resultant remapping effects were long lasting, such that place field representations during recovery were more similar to inactivated sessions than to baseline and these changes remained for multiple recording days. The authors suggest that the MEA is instead involved in selecting the active population of neurons within the hippocampus for a given environment. These findings combined with our own suggest that the LEA, but not the MEA, is involved in selection of cues an animal uses for spatial orientation.

In summary, our findings suggest that the hippocampus and the LEA differentiate cues that are useful for navigation from those that are not. We previously showed that the hippocampus processes 2D landmarks and 2D objects differently (Scaplen et al., 2014). In the present study, we showed that LEA inactivation had qualitatively different impact on cues that are useful for navigation vs. cues that may not be useful for navigation but might be available for associative learning. These data provide evidence that the LEA is involved in modulating how the dorsal hippocampus utilizes visual environmental cues. Future research is needed to further elucidate the role of the LEA in the hippocampal dependent use of environmental cues for navigation and associative learning.

## Author Contributions

KMS conceived and designed experiments; acquired, analyzed, and interpreted data; and wrote the article. RNR contributed to data acquisition and analysis. NN contributed to data analysis and interpretation. OJA contributed to data analysis, interpretation and participated in revising the article. RDB conceived and designed experiments; contributed to data analysis and interpretation and wrote the article.

## Conflict of Interest Statement

The authors declare that the research was conducted in the absence of any commercial or financial relationships that could be construed as a potential conflict of interest.

## References

[B1] Agster K. L. Burwell R. D. (2009). Cortical efferents of the perirhinal, postrhinal, and entorhinal cortices of the rat. Hippocampus 19, 1159–1186. 10.1002/hipo.20578 19360714 PMC3066185

[B2] Agster K. L. Burwell R. D. (2013). Hippocampal and subicular efferents and afferents of the perirhinal, postrhinal and entorhinal cortices of the rat. Behav. Brain Res. 254, 50–64. 10.1016/j.bbr.2013.07.005 23872326 PMC3792719

[B3] Agster K. L. Tomas Pereira I. Saddoris M. P. Burwell R. D. (2016). Subcortical connections of the perirhinal, postrhinal and entorhinal cortices of the rat. II. efferents. Hippocampus 26, 1213–1230. 10.1002/hipo.22600 27101786 PMC5070461

[B4] Ahmed O. J. Mehta M. R. (2009). The hippocampal rate code: anatomy, physiology and theory. Trends Neurosci. 32, 329–338. 10.1016/j.tins.2009.01.009 19406485 PMC3066563

[B100] Ahmed O. J. Mehta M. R. (2012). Running speed alters the frequency of hippocampal gamma oscillations. J. Neurosci. 32, 7373–7383. 10.1523/JNEUROSCI.5110-11.2012 22623683 PMC3366345

[B5] Allen T. A. Narayanan N. S. Kholodar-Smith D. B. Zhao Y. Laubach M. Brown T. H. (2008). Imaging the spread of reversible brain inactivations using fluorescent muscimol. J. Neurosci. Methods 171, 30–38. 10.1016/j.jneumeth.2008.01.033 18377997 PMC2440580

[B6] Blackstad T. W. (1956). Commissural connections of the hippocampal region in the rat, with special reference to their mode of termination. J. Comp. Neurol. 105, 417–537. 10.1002/cne.901050305 13385382

[B7] Burke S. N. Maurer A. P. Nematollahi S. Uprety A. R. Wallace J. L. Barnes C. A. (2011). The influence of objects on place field expression and size in distal hippocampal CA1. Hippocampus 21, 783–801. 10.1002/hipo.20929 21365714 PMC3314262

[B8] Burwell R. D. Amaral D. G. (1998). Cortical afferents of the perirhinal, postrhinal, and entorhinal cortices of the rat. J. Comp. Neurol. 398, 179–205. 10.1002/(SICI)1096-9861(19980824)398:2<179::AID-CNE3>3.0.CO;2-Y 9700566

[B9] Burwell R. D. Hafeman D. M. (2003). Positional firing properties of postrhinal cortex neurons. Neuroscience 119, 577–588. 10.1016/s0306-4522(03)00160-x 12770570

[B10] Buzsáki G. (2002). Theta oscillations in the hippocampus. Neuron 33, 325–340. 10.1016/s0896-6273(02)00586-x 11832222

[B11] Cho H. J. Sung Y. H. Lee S. H. Chung J. Y. Kang J. M. Yi J. W. (2013). Isoflurane induces transient anterograde amnesia through suppression of brain-derived neurotrophic factor in hippocampus. J. Korean Neurosurg. Soc. 53, 139–144. 10.3340/jkns.2013.53.3.139 23634262 PMC3638265

[B13] Deshmukh S. S. Johnson J. L. Knierim J. J. (2012). Perirhinal cortex represents nonspatial, but not spatial, information in rats foraging in the presence of objects: comparison with lateral entorhinal cortex. Hippocampus 22, 2045–2058. 10.1002/hipo.22046 22987681 PMC3870144

[B12] Deshmukh S. S. Knierim J. J. (2011). Representation of non-spatial and spatial information in the lateral entorhinal cortex. Front. Behav. Neurosci. 5:69. 10.3389/fnbeh.2011.00069 22065409 PMC3203372

[B14] Fidalgo A. R. Cibelli M. White J. P. Nagy I. Wan Y. Ma D. (2012). Isoflurane causes neocortical but not hippocampal-dependent memory impairment in mice. Acta Anaesthesiol. Scand. 56, 1052–1057. 10.1111/j.1399-6576.2012.02691.x 22471713

[B15] Furtak S. C. Ahmed O. J. Burwell R. D. (2012). Single neuron activity and theta modulation in postrhinal cortex during visual object discrimination. Neuron 76, 976–988. 10.1016/j.neuron.2012.10.039 23217745 PMC3523310

[B16] Furtak S. C. Cho C. E. Kerr K. M. Barredo J. L. Alleyne J. E. Patterson Y. R.. (2009). The floor projection maze: a novel behavioral apparatus for presenting visual stimuli to rats. J. Neurosci. Methods 181, 82–88. 10.1016/j.jneumeth.2009.04.023 19422855 PMC2883467

[B17] Fyhn M. Molden S. Witter M. P. Moser E. I. Moser M.-B. (2004). Spatial representation in the entorhinal cortex. Science 305, 1258–1264. 10.1126/science.1099901 15333832

[B18] Hafting T. Fyhn M. Molden S. Moser M.-B. Moser E. I. (2005). Microstructure of a spatial map in the entorhinal cortex. Nature 436, 801–806. 10.1038/nature03721 15965463

[B19] Hargreaves E. L. Rao G. Lee I. Knierim J. J. (2005). Major dissociation between medial and lateral entorhinal input to dorsal hippocampus. Science 308, 1792–1794. 10.1126/science.1110449 15961670

[B20] Henriksen E. J. Colgin L. L. Barnes C. A. Witter M. P. Moser M.-B. Moser E. I. (2010). Spatial representation along the proximodistal axis of CA1. Neuron 68, 127–137. 10.1016/j.neuron.2010.08.042 20920796 PMC3093538

[B21] Hikosaka O. Wurtz R. H. (1985). Modification of saccadic eye movements by GABA-related substances. I. Effect of muscimol and bicuculline in monkey superior colliculus. J. Neurophysiol. 53, 266–291. 2983037 10.1152/jn.1985.53.1.266

[B22] Hunsaker M. R. Chen V. Tran G. T. Kesner R. P. (2013). The medial and lateral entorhinal cortex both contribute to contextual and item recognition memory: a test of the binding of items and context model. Hippocampus 23, 380–391. 10.1002/hipo.22097 23436324

[B23] Jacobson T. K. Ho J. W. Kent B. W. Yang F. C. Burwell R. D. (2014). Automated visual cognitive tasks for recording neural activity using a floor projection maze. J. Vis. Exp. 84:e51316. 10.3791/51316 24638057 PMC4130232

[B24] Kerr K. M. Agster K. L. Furtak S. C. Burwell R. D. (2007). Functional neuroanatomy of the parahippocampal region: the lateral and medial entorhinal areas. Hippocampus 17, 697–708. 10.1002/hipo.20315 17607757

[B25] Knierim J. J. Lee I. Hargreaves E. L. (2006). Hippocampal place cells: parallel input streams, subregional processing and implications for episodic memory. Hippocampus 16, 755–764. 10.1002/hipo.20203 16883558

[B26] Krieg W. J. (1946a). Connections of the cerebral cortex. the albino rat. structure of the cortical areas. J. Comp. Neurol. 84, 277–323. 10.1002/cne.900840302 20991808

[B27] Krieg W. J. (1946b). Connections of the cerebral cortex; the albino rat; topography of the cortical areas. J. Comp. Neurol. 84, 221–275. 10.1002/cne.900840205 20982805

[B28] Krupa D. J. Ghazanfar A. A. Nicolelis M. A. (1999). Immediate thalamic sensory plasticity depends on corticothalamic feedback. Proc. Natl. Acad. Sci. U S A 96, 8200–8205. 10.1073/pnas.96.14.8200 10393972 PMC22212

[B29] Lee I. Knierim J. J. (2007). The relationship between the field-shifting phenomenon and representational coherence of place cells in CA1 and CA3 in a cue-altered environment. Learn. Mem. 14, 807–815. 10.1101/lm.706207 18007023 PMC2080582

[B31] Leutgeb S. Leutgeb J. K. Barnes C. A. Moser E. I. McNaughton B. L. Moser M.-B. (2005). Independent codes for spatial and episodic memory in hippocampal neuronal ensembles. Science 309, 619–623. 10.1126/science.1114037 16040709

[B30] Leutgeb J. K. Leutgeb S. Treves A. Meyer R. Barnes C. A. McNaughton B. L.. (2005). Progressive transformation of hippocampal neuronal representations in “morphed” environments. Neuron 48, 345–358. 10.1016/j.neuron.2005.09.007 16242413

[B32] Lin D. Zuo Z. (2011). Isoflurane induces hippocampal cell injury and cognitive impairments in adult rats. Neuropharmacology 61, 1354–1359. 10.1016/j.neuropharm.2011.08.011 21864548 PMC3189329

[B33] Markus E. J. Barnes C. A. McNaughton B. L. Gladden V. L. Skaggs W. E. (1994). Spatial information content and reliability of hippocampal CA1 neurons: effects of visual input. Hippocampus 4, 410–421. 10.1002/hipo.450040404 7874233

[B34] Markus E. J. Qin Y. L. Leonard B. Skaggs W. E. McNaughton B. L. Barnes C. A. (1995). Interactions between location and task affect the spatial and directional firing of hippocampal neurons. J. Neurosci. 15, 7079–7094. 7472463 10.1523/JNEUROSCI.15-11-07079.1995 PMC6578055

[B36] Moser M.-B. Moser E. I. (1998). Functional differentiation in the hippocampus. Hippocampus 8, 608–619. 10.1002/(SICI)1098-1063(1998)8:6<608::AID-HIPO3>3.0.CO;2-7 9882018

[B35] Moser E. I. Moser M.-B. (2008). A metric for space. Hippocampus 18, 1142–1156. 10.1002/hipo.20483 19021254

[B37] Neunuebel J. P. Yoganarasimha D. Rao G. Knierim J. J. (2013). Conflicts between local and global spatial frameworks dissociate neural representations of the lateral and medial entorhinal cortex. J. Neurosci. 33, 9246–9258. 10.1523/JNEUROSCI.2897-13.2013 23719794 PMC3747988

[B40] Parron C. Poucet B. Save E. (2004). Entorhinal cortex lesions impair the use of distal but not proximal landmarks during place navigation in the rat. Behav. Brain Res. 154, 345–352. 10.1016/j.bbr.2004.03.006 15313022

[B38] Parron C. Save E. (2004a). Comparison of the effects of entorhinal and retrosplenial cortical lesions on habituation, reaction to spatial and non-spatial changes during object exploration in the rat. Neurobiol. Learn. Mem. 82, 1–11. 10.1016/j.nlm.2004.03.004 15183166

[B39] Parron C. Save E. (2004b). Evidence for entorhinal and parietal cortices involvement in path integration in the rat. Exp. Brain Res. 159, 349–359. 10.1007/s00221-004-1960-8 15526193

[B41] Rueckemann J. W. DiMauro A. J. Rangel L. M. Han X. Boyden E. S. Eichenbaum H. (2016). Transient optogenetic inactivation of the medial entorhinal cortex biases the active population of hippocampal neurons. Hippocampus 26, 246–260. 10.1002/hipo.22519 26299904 PMC4718858

[B42] Scaplen K. M. Gulati A. A. Heimer-McGinn V. L. Burwell R. D. (2014). Objects and landmarks: hippocampal place cells respond differently to manipulations of visual cues depending on size, perspective, and experience. Hippocampus 24, 1287–1299. 10.1002/hipo.22331 25045010 PMC5615844

[B43] Skaggs W. McNaughton B. Gothard K. Markus E. (1993). An Information Theoretic Approach to Deciphering the Hippocampal Code. San Mateo, CA: Morgan Kaufmann.

[B44] Steward O. Scoville S. A. (1976). Retrograde labeling of central nervous pathways with tritiated or Evans blue-labeled bovine serum albumin. Neurosci. Lett. 3, 191–196. 10.1016/0304-3940(76)90072-0 19604885

[B45] Stranahan A. M. Salas-Vega S. Jiam N. T. Gallagher M. (2011). Interference with reelin signaling in the lateral entorhinal cortex impairs spatial memory. Neurobiol. Learn. Mem. 96, 150–155. 10.1016/j.nlm.2011.03.009 21492744 PMC3148331

[B46] Tanninen S. E. Morrissey M. D. Takehara-Nishiuchi K. (2013). Unilateral lateral entorhinal inactivation impairs memory expression in trace eyeblink conditioning. PLoS One 8:e84543. 10.1371/journal.pone.0084543 24367674 PMC3868607

[B47] Tomás Pereira I. Agster K. L. Burwell R. D. (2016). Subcortical connections of the perirhinal, postrhinal, and entorhinal cortices of the rat. I. afferents. Hippocampus 26, 1189–1212. 10.1002/hipo.22603 27119220 PMC5070464

[B48] Tsao A. Moser M.-B. Moser E. I. (2013). Traces of experience in the lateral entorhinal cortex. Curr. Biol. 23, 399–405. 10.1016/j.cub.2013.01.036 23434282

[B49] Van Cauter T. Camon J. Alvernhe A. Elduayen C. Sargolini F. Save E. (2013). Distinct roles of medial and lateral entorhinal cortex in spatial cognition. Cereb. Cortex 23, 451–459. 10.1093/cercor/bhs033 22357665

[B50] van Groen T. Miettinen P. Kadish I. (2003). The entorhinal cortex of the mouse: organization of the projection to the hippocampal formation. Hippocampus 13, 133–149. 10.1002/hipo.10037 12625464

[B51] Wilson D. I. Langston R. F. Schlesiger M. I. Wagner M. Watanabe S. Ainge J. A. (2013). Lateral entorhinal cortex is critical for novel object-context recognition. Hippocampus 23, 352–366. 10.1002/hipo.22095 23389958 PMC3648979

[B52] Wood E. R. Dudchenko P. A. Robitsek R. J. Eichenbaum H. (2000). Hippocampal neurons encode information about different types of memory episodes occurring in the same location. Neuron 27, 623–633. 10.1016/s0896-6273(00)00071-4 11055443

[B53] Wyss J. M. (1981). An autoradiographic study of the efferent connections of the entorhinal cortex in the rat. J. Comp. Neurol. 199, 495–512. 10.1002/cne.901990405 6168668

